# Mitoxantrone and ametantrone induce interstrand cross-links in DNA of tumour cells

**DOI:** 10.1054/bjoc.1999.1095

**Published:** 2000-03-06

**Authors:** A Skladanowski, J Konopa

**Affiliations:** Department of Pharmaceutical Technology and Biochemistry, Technical University of Gdańsk, Narutowicza St 11/12, Gdańsk, 80–952, Poland

**Keywords:** aminoanthraquinones, mitoxantrone, mechanism of action, DNA cross-linking, covalent binding, metabolism

## Abstract

We show here that mitoxantrone and ametantrone induce interstrand DNA cross-links in HeLa S_3_cells. These cross-links were observed only in cellular system suggesting that metabolism of the drugs is a necessary step leading to DNA cross-linking. Biologically inactive analogue of mitoxantrone, compound NSC 321458, did not induce cross-links in DNA of tumour cells which suggests that DNA cross-linking is associated with the cytotoxic and anti-tumour activity of these compounds. © 2000 Cancer Research Campaign


*British Journal of Cancer* (2000) **82**(7), 1300–1304
© 2000 Cancer Research Campaign
DOI: 10.1054/ bjoc.1999.1095, available online at http://www.idealibrary.com on IDEAL®

### Mitoxantrone and ametantrone induce interstrand cross-links in DNA of tumour cells

**A Skladanowski and J Konopa**

Department of Pharmaceutical Technology and Biochemistry, Technical University of Gdan´sk, Narutowicza St 11/12, 80–952 Gdan´sk, Poland

**Summary** We show here that mitoxantrone and ametantrone induce interstrand DNA cross-links in HeLa $S_3$ cells. These cross-links were observed only in cellular system suggesting that metabolism of the drugs is a necessary step leading to DNA cross-linking. Biologically inactive analogue of mitoxantrone, compound NSC 321458, did not induce cross-links in DNA of tumour cells which suggests that DNA cross-linking is associated with the cytotoxic and anti-tumour activity of these compounds. © 2000 Cancer Research Campaign

**Keywords**: aminoanthraquinones; mitoxantrone; mechanism of action; DNA cross-linking; covalent binding; metabolism

Mitoxantrone is an anti-tumour drug, developed by a rational modification of the parent 1,4-dehydroxy analogue, ametantrone (for review see Cheng et al, 1989). The mechanism of action of mitoxantrone and other aminoalkyl anthracenediones involves multiple effects on cellular DNA but strong DNA binding through intercalation is believed to be responsible for the pharmacological activity of these agents (for review see Cheng et al, 1989; Fry, 1991). Mitoxantrone also inhibits DNA topoisomerase II and targetting of this enzyme contributes to the anti-tumour effect of this drug at least in some cellular systems (Smith et al, 1990 and references therein). However, several mitoxantrone analogues have been identified which do not inhibit topoisomerase II but still exhibit high anti-tumour activity (Glisson et al, 1990). Structure–activity relationship studies showed the crucial role of diaminoalkyl group in side chains of anthracenediones for the biological activity of these compounds (for review see Cheng et al, 1989) but the importance of this group for any of the proposed mechanisms of action of anthracenediones is not clear.

Our previous studies have shown that anthracyclines, such as doxorubicin and daunorubicin, induce DNA cross-links in tumour cells (Konopa, 1983; Skladanowski and Konopa, 1994*a*) and the induction of DNA cross-links seems to be relevant for biological ativity of these drugs (Skladanowski and Konopa, 1994*b*). Anthracenediones may be regarded as structural analogues of anthracyclines, and mitoxantrone and ametantrone are readily metabolized both in vitro and in vivo (Ehninger et al, 1990 and references therein) to chemically reactive species which bind DNA (Reszka et al, 1989; Mewes et al, 1993). The present study explored the possibility that mitoxantrone and ametantrone may form interstrand cross-links in DNA of tumour cells.

### MATERIALS AND METHODS

### Chemicals

All reagents were from Sigma (Poznan, Poland); [$^{14}$C]-thymidine was from Amersham International (Amersham, UK.).

*Received 9 June 1999*
*Revised 11 November 1999*
*Accepted 17 November 1999*

*Correspondence to:* J Konopa

### Drugs

Mitoxantrone and ametantrone were synthesized in our Department, and the biologically inactive analogue of mitoxantrone NSC 321458 (for chemical structures see Figure 1) was provided by the National Cancer Institute (Natural Products Branch, Bethesda, MD, USA) by courtesy of Dr Matthew Suffness.

### Cell culture and media

HeLa $S_3$ cells, the media, glutamine and fetal calf serum (FCS) were from Gibco Europe Ltd (Paisley, UK). Antibiotics were from Serva (Heidelberg, Germany). Cells were grown in a monolayer culture in MEM (minimal essential media) supplemented with 5% FCS and antibiotics. The cytotoxicity of anthracenediones was determined after 3-h drug treatment followed by 96-h post-incubation by the MTT 3-(4,5-dimethylthiazal-2-yl)-2,5-diphenyltetrazolium bromide) assay. The $EC_{50}$ dose (drug concentration resulting in 50% inhibition of MTT dye formation, compared to

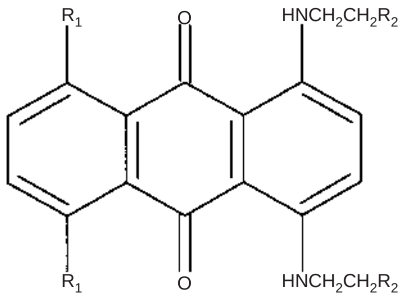

| | $R_1$ | $R_2$ |
|---|---|---|
| Mitoxantrone | OH | $HNCH_2CH_2OH$ |
| Ametantrone | H | $HNCH_2CH_2OH$ |
| NSC 321458 | OH | $CH_2CH_2CH_2OH$ |

**Figure 1** Chemical structure of the studied anthracenediones

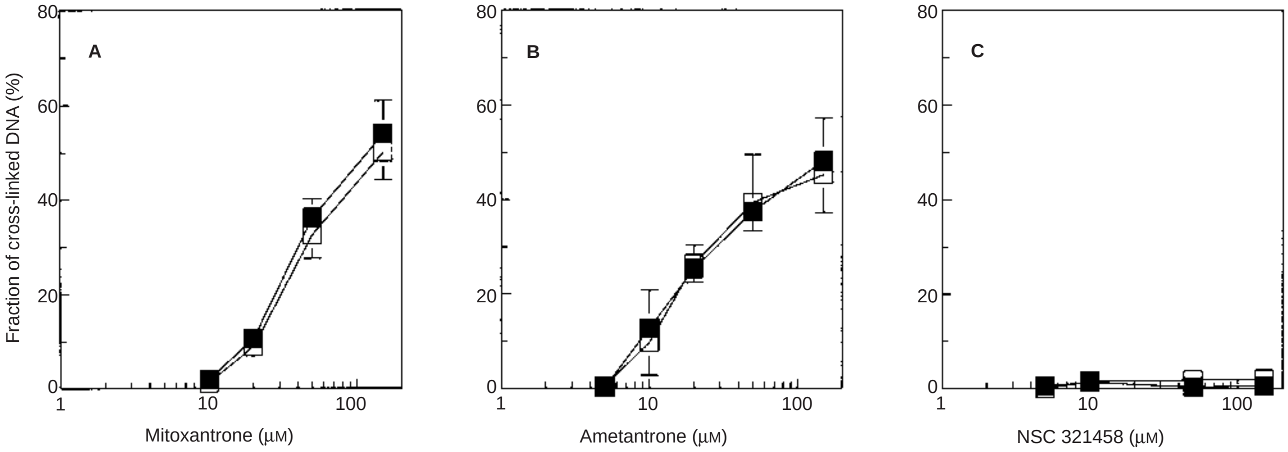

**Figure 2** DNA cross-linking induced by mitoxantrone (**A**), ametantrone (**B**) and NSC 321458 (**C**). HeLa $S_3$ cells were incubated with the compounds for 3 h and fractions of cross-linked DNA were determined by the method described in Materials and Methods without (closed symbols) and with (open symbols) protein digestion. *Points*, mean from three independent experiments; *bars*, s.d controls) was calculated directly from semi-logarithmic dose–response curves.

### Determination of interstrand DNA cross-links

HeLa S3 cells ($2.5 \times 10^6$ cells 10 $ml^{-1}$) were exposed to various concentrations of drugs for 3 h at 37°C. Interstrand DNA cross-links were determined by the procedure described in detail previously (Skladanowski and Konopa, 1994*a*). Briefly, after lysis of cells in lysis buffer (6.8 M sodium perchlorate, 1 mM EDTA, 0.2% lauroyl sarcosine, 20% v/v methanol, pH 7) cellular DNA was denatured by heating samples at 50°C for 30 min and allowing them to renature by rapid dilution with 20 ml of ice-cold acetate buffer (0.04 M sodium acetate, 0.4 M sodium chloride, 5 mM zinc acetate, pH 4.4) and cooling in methanol–ice mixture (–18°C, 1 min). The percentage of interstrand cross-linked DNA was determined using nuclease $S_1$ assay as described previously (Konopa, 1983). The fraction of renatured double-stranded DNA from untreated cells did not exceed 10–15%. The fraction of cross-linked DNA was calculated according to the formula:

$$F_{CR} = \frac{RF_{treat} - RF_{contr}}{100 - RF_{contr}} \times 100\%$$

where $RF_{treat}$ and $RF_{contr}$ are fractions of renatured DNA in drug-treated and non-treated cells respectively.

For cell-free system studies, drugs were added directly to the cell lysates prepared as described above and incubated protected from light at 37°C for 3 h. The cellular DNA was then denatured and renatured and fractions of cross-linked DNA were determined as described above. For thermal denaturation, samples were heated at 95°C, 15 min and cooled in methanol–ice bath for 2 min. For alkaline denaturation, 0.12 ml 1 M sodium hydroxide was gently added to give 30 mM final concentration and after 1-h incubation in the dark at room temperature the samples were neutralized with 0.12 ml 1 M hydrochloric acid (HCl) before DNA cross-linking measurements.

In some experiments, cell lysates were deproteinized before DNA denaturation. Following treatment with the drugs, cells were suspended in 400 μl of lysing buffer (50 mM Tris–HCI, pH 7.5, 5 mM EDTA, 0.5% lauroyl sarcosine, 0.5 mg $ml^{-1}$ proteinase K) and incubated at 37°C for 2 h. Then 3.6 ml lysing solution (see above) was added and lysis was carried out at 37°C for an additional 1 h and DNA cross-links were determined as described above.

**Table 1** Cytotoxic activity against HeLa $S_3$ cells and DNA cross-linking potency of the studied anthracenediones

| Compound | $EC_{50}$ (μM)[a] | $DPC_{max}$[b] | $C_0$ (μM)[c] |
|---|---|---|---|
| Mitoxantrone | 0.2 | 1.0 | 9.8 |
| Ametantrone | 2.7 | 0.47 | 4.1 |
| NSC 321458 | > 100 | 0.24 | – |

[a]Concentration inhibiting growth of HeLa $S_3$ cells by 50% compared to non-treated cells. [b]Relative level of DNA–protein complexes induced by drugs (mitoxantrone = 1). [c]Concentration at which the first DNA cross-link could be detected.

### Topoisomerase II-associated DNA damage in intact cells

DNA–protein complexes were quantified by the potassium chloride (KCl)-SDS (sodium dodecyl sulphate) co-precipitation assay as described (Zwelling et al, 1989). Data are expressed as the ratio of [$^3$H]DNA to [$^{14}$C]protein, with protein being an internal standard for the exact number of cells used.

### RESULTS

### Interstrand DNA cross-linking in DNA of tumour cells

Exposure of HeLa $S_3$ to either mitoxantrone or ametantrone resulted in an increase in the fractions of cross-linked DNA ($F_{CR}$) with increasing concentration of the drugs (Figure 2, A and B, closed symbols). A close structural analogue of mitoxantrone, compound NSC 321458, did not induce any interstrand DNA crosslinking in HeLa $S_3$ cells even at concentrations as high as 150 μM (Figure 2C, closed symbols). This compound lacks the distal nitrogen atoms in the side chains (compare chemical

© 2000 Cancer Research Campaign 

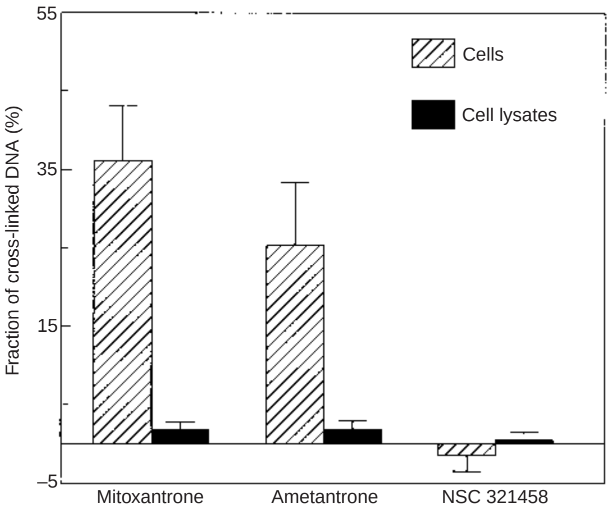

**Figure 3** Fractions of cross-linked DNA determined in HeLa $S_3$ cells (hatched bars) and cell lysates (solid bars) treated with the studied anthracenediones. The cells and cell lysates were incubated with anthracenediones at 50 µM for 3 h at 37°C and fractions of cross-linked DNA were determined as described in Materials and Methods

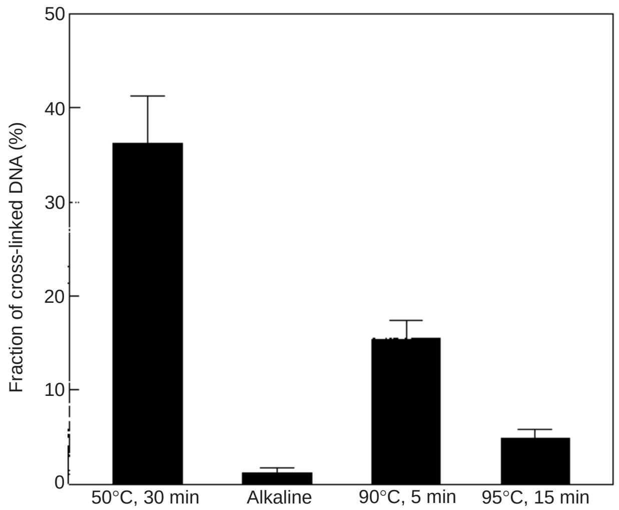

**Figure 5** Thermal and alkaline instability of DNA cross-links induced by mitoxantrone in HeLa $S_3$. The cells were treated with the compound at 50 µM for 3 h at 37°C and fractions of cross-linked DNA were determined as described in Materials and Methods with various denaturation conditions

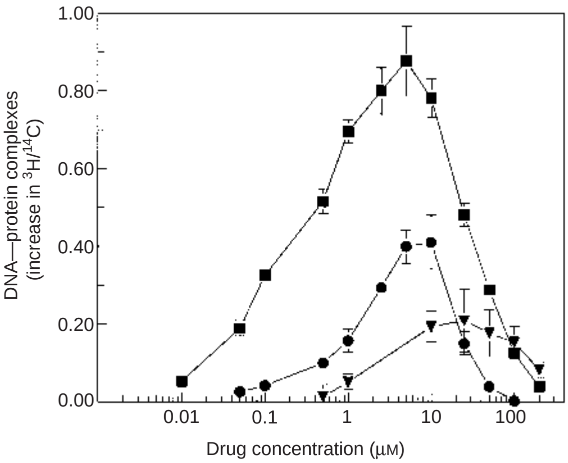

**Figure 4** DNA–protein complexes induced by anthracenediones in HeLa S3 cells. Cells were exposed for 3 h to different doses of mitoxantrone (■), ametantrone (●) and NSC321458 (▼) and the level of DNA–protein complexes was determined by the K–SDS co-precipitation assay structures in Figure 1) and is not cytotoxic toward HeLa $S_3$ cells ($EC_{50}$ > 100 µM). To characterize DNA cross-linking potency of the studied compounds, we calculated concentration at which the first cross-link could be detected ($C_0$ concentration) by our method. This was done by a linear fit of the relationship between drug concentration and fraction of cross-linked DNA ($F_{CR}$) and extrapolation to intercept $C_0$, at 0% of $F_{CR}$. The $C_0$ values and cytotoxic activity (the $EC_{50}$ concentration), determined in the same conditions as DNA cross–linking, for all anthracenediones studied are shown in Table 1.

### The lack of DNA cross-linking in cell-free system

Mitoxantrone and ametantrone show high affinity binding to DNA and increased renaturation of DNA from cells treated with anthracenediones may result from stabilization of DNA structure by these drugs due to stable physicochemical intercalative complexes with DNA. To distinguish between physicochemical and covalent cross-linking of DNA from drug-treated cells, cell lysates from untreated HeLa $S_3$ cells were incubated with the studied compounds at concentration (50 µM) which produced significant DNA cross-linking in whole cells. Fractions of cross-linked DNA, determined when the studied anthracenediones were added directly to the cell lysates for 3 h at 37°C and then processed as for whole cells, were very low compared to those obtained for DNA from cells treated with the same concentrations of the drugs and the same time of treatment (Figure 3). It suggests that strong physicochemical binding of the studied anthracenediones to DNA is not responsible for enhanced renaturability of DNA from treated cells.

### Stabilization of DNA–protein complexes by anthracenediones

Mitoxantrone and ametantrone stabilize topoisomerase II–DNA cleavable complexes (Smith et al, 1990 and this study). Therefore, it was possible that increased renaturation of DNA from cells treated with these compounds stems from DNA–topoisomerase II complexes which may survive mild denaturation conditions. To clarify this point, we determined the level of DNA–protein complexes induced by studied compounds by the K–SDS co-precipitation assay. As shown in Figure 4, all the anthracenediones induced DNA–protein complexes with characteristic bell-shaped dose–response curves. Interestingly, even a biologically inactive derivative, NSC 321458, produced measurable level of DNA–protein complexes (about twofold lower than ametantrone) although this compound is only marginally cytotoxic (> 80% viability) at concentrations up to 100 µM. The effect of stabilization of DNA–protein complexes by studied drugs on DNA renaturation was assessed by including deproteinization step in the procedure for DNA cross-links measurements. When proteins in cellular lysates were digested by proteinase K before DNA denaturation, increased fraction of renaturable DNA from cells treated

 © 2000 Cancer Research Campaign with drugs was still observed for mitoxantrone and ametantrone, and was quite similar to the results obtained when protein digestion was omitted (Figure 2, open symbols).

### Thermal and alkaline instability of DNA cross-links

No DNA cross-links could be observed when DNA from cells treated with mitoxantrone was denatured in alkaline conditions (pH 12, 1 h); however, they partially resisted elevated temperature (Figure 5). The $F_{CR}$ values obtained for DNA from cells treated with 50 μM mitoxantrone and denatured at 95°C for 5 min are about 50% the value obtained when DNA denaturation was performed at lower temperature according to the method used throughout these studies (50°C, 30 min), whereas only marginal DNA cross-linking was observed when thermal denaturation of DNA was prolonged to 15 min (Figure 5). Similar results were obtained for ametantrone (data not shown).

### DISCUSSION

We have found that mitoxantrone and ametantrone produced DNA cross-links in HeLa $S_3$ cells in a concentration-dependent manner. First, DNA cross-links induced by mitoxantrone and ametantrone could be observed at concentrations of about 5–10 μM. Previous studies showed that peak plasma concentration of mitoxantrone in patients treated with this drug is about 10 μM (5–13 μM) and remains constant for about 20 h after drug treatment (Sundman-Engberg et al, 1993). It follows that DNA cross-links formed by anthracenediones could be detected by our method at concentrations close to those physiologically achieveable.

Mitoxantrone and ametantrone did not produce any DNA cross-linking when added directly to cell lysates, in which cellular enzymes were inactivated. From this observation two conclusions can be drawn. First, the increased renaturation of DNA from cells treated with aminoanthraquinones was not the effect of their stabilization of DNA structure by intercalation, and second, that metabolic activation of these two compounds is required for covalent binding and cross-linking of cellular DNA. Mitoxantrone and ametantrone possess no reactive groups which bind covalently to DNA but these compounds have been shown to be readily metabolized in vitro and in whole cells (see Ehninger et al, 1990 and references therein). Oxidation of mitoxantrone results in covalent binding of the drug to DNA and thiols (Reszka et al, 1989; Mewes et al, 1993; Dackiewicz et al, 1995) and intramolecular cross-linking of plasmid DNA in vitro (Fisher and Patterson, 1990).

A close structural analogue of mitoxantrone, NSC 321458, which is biologically inactive, did not produce any DNA cross-links in HeLa $S_3$ cells. In contrast, this compound stimulated low levels of DNA–protein complexes in whole cells, only one-half of those observed for ametantrone. It would suggest that the effect of NSC 321458 on topoisomerase II-associated DNA complexes cannot fully explain the lack of biological activity of this compound toward HeLa S3 cells. NSC 321458 differs from mitoxantrone in the lack of the distal amino groups in the side chains. It may suggest that alkyldiamine residues are involved in covalent binding and DNA cross-linking by mitoxantrone and ametantrone. Our preliminary results showed that activation of mitoxantrone by rat liver microsomes leads to formation of reactive hydroxylamine residues (unpublished results). Therefore, covalent binding to DNA by enzymatically activated alkylamine groups could explain the essential role of these moieties for biological activity of aminoanthraquinones shown by several authors (see Cheng et al, 1989 and references therein).

The fact that alkyldiamine residue may be of the primary importance for biological activity and DNA crosslinking ability of mitoxantrone suggested to us that attachment of alkyldiamine moiety to other planar polycyclic chromophore favouring intercalative binding to DNA should yield new anti-tumour compounds. The role of the planar nucleus would be to position of the compound within the DNA structure, and structural modifications of the chromophore system should lead to enhanced reactivity of alkyldiamine residue during metabolic activation by cellular enzymes. Accordingly, several new groups of very active anti-tumour compounds were synthesized in our laboratory, derivatives of imidazoacridone (Cholody et al, 1990a) and triazoloacridone (Cholody et al, 1990*b*).

We observed that ametantrone is a more potent DNA cross-linking agent than mitoxantrone although the latter is more than ten times more cytotoxic toward HeLa $S_3$ cells (compare $C_0$ and $EC_{50}$ values for ametantrone and mitoxantrone in Table 1). On the other hand, ametantrone produces approximately twofold less DNA–protein complexes than mitoxantrone (Figure 4 and Table 1). We favour the possibility that overlapping protein-associated DNA breaks lower renaturation fraction of DNA at concentrations where both DNA cross-linking and stabilization of DNA–protein complexes is observed. It would lead to a higher $C_0$ value for mitoxantrone compared to ametantrone. Further studies are required to clarify this notion.

DNA cross-links produced by mitoxantrone and ametantrone resisted only a short exposure to elevated temperature, therefore they are unlikely to be detected by the methods based on thermal denaturation of DNA. Additionally, the cross-links were disrupted at alkaline pH (pH 12, 1 h) and therefore could not be detected by alkaline elution procedures where DNA is exposed to alkali for a prolonged period of time (pH 12, about 12 h). For this reason, in this study we used a new mild procedure developed in our laboratory (Skladanowski and Konopa, 1994*a*). Validity of this method was proved by measuring DNA cross-linking induced by mitomycin C, a classical bifunctional DNA alkylator (data not shown).

In conclusion, we here show that two aminoanthraquinone drugs, mitoxantrone and ametantrone, induce covalent DNA cross-links in HeLa $S_3$ cells at concentrations close to those clinically achievable. The formation of DNA cross-links by these two drugs was not observed in cell lysated with inactivated cellular enzymes, suggesting that metabolic activation is necessary for DNA cross-linking to occur. The significance of DNA cross-linking by mitoxantrone and ametantrone for their biological activity is not clear at this point and is a subject of ongoing studies in our laboratory.

### ACKNOWLEDGEMENTS

This work was supported by the Polish Committee for Scientific Research (KBN), grant no 4 0109 91 01.

### REFERENCES

Cheng CC and Zee-Cheng RKY (1989) The design, synthesis and development of a new class of potent antineoplastic anthraquinones. In: *Progress in Medicinal Chemistry*, Ellis GP and West GB (eds), pp. 83–118. Elsevier Science Publishers BV: New York

*© 2000 Cancer Research Campaign* 

Cholody M, Martelli S, Paradziej-Lukowicz J and Konopa J (1990*a*) 5-[(aminoalkyl) amino]imidazo[4,5,1-*de*]acridin-6-ones as a novel class of antineoplastic agents. *J Med Chem* **33**: 49–52

Cholody M, Martelli S and Konopa J (1990*b*) 8-substituted 5-[(aminoalkyl)amino] triazolo[4,5,1-*de*]acridin-6-ones as potential antineoplastic agents. Synthesis and biological activity. *J Med Chem* **33**: 2852–2856

Dackiewicz P, Skladanowski A and Konopa J (1995) $^{32}$P-postlabeling analysis of adducts formed by mitoxantrone and ametantrone with DNA and homopolydeoxynucleotides after enzymatic activation. *Chem Biol Interactions* **98**: 153–166

Ehninger G, Schuler U, Proksch B, Zeller K-P and Blanz J (1990) Pharmacokinetics and metabolism of mitoxantrone. *Clin Pharmacokinet* **18**(5): 365–380

Fisher GR and Patterson LH (1990) DNA strand breakage by peroxidase-activated mitoxantrone. *J Pharm Pharmacol* **43**: 65–68

Fry DW (1991) Biochemical pharmacology of anthracenediones and anthrapyrazoles. *Pharmac Ther* **52**: 109–125

Glisson BS, Killary AM, Merta P, Ross WE, Siciliano J and Siciliano MJ (1990) Dissociation of cytotoxicity and DNA cleavage activity induced by topoisomerase II-reactive intercalating agents in hamster–human somatic cell hybrids. *Cancer Chemother Pharmacol* **31**: 131–138

Konopa J (1983) Adriamycin and daunomycin induce interstrand DNA cross-links in HeLa S3 cells. *Biochem Biophys Res Commun* **110**: 819–826

Mewes K, Blanz J, Ehninger G, Gebhardt R and Zeller K-P (1993) Cytochrome P450-induced cytotoxicity of mitoxantrone by formation of electrophophilic intermediates. *Cancer Res* **53**: 5135–5142

Reszka K, Hartley JH, Kolodziejczyk P and Lown JW (1989) Interaction of the peroxidase-derived metabolite of mitoxantrone with nucleic acids. *Biochem Pharmacol* **38**: 4253–4260

Skladanowski A and Konopa J (1994*a*) Interstrand DNA cross-linking induced by anthracyclines in tumor cells. *Biochem Pharmacol* **47**: 2269–2278

Skladanowski A and Konopa J (1994*b*) Relevance of interstrand DNA cross-linking induced by anthracyclines for their biological activity. *Biochem Pharmacol* **47**: 2279–2287

Smith PJ, Morgan SA, Fox ME and Watson JV (1990) Mitoxantrone–DNA binding and the induction of toposiomerase II associated DNA damage in multidrug resistant small cell lung cancer cells. *Biochem Pharmacol* **40**: 2069–2078

Sundman-Engberg B, Tidefelt U, Gruber A and Christer P (1993) Intracellular concentrations of mitoxantrone in leukemic cells in vitro and in vivo. *Leukemia Res* **17**: 347–352

Zwelling LA, Hinds M, Chan D, Mayes J, Sie KL, Parker E, Silberman L, Radcliffe A, Beran M and Blick M (1989) Characterization of an amsacrine-resistant line of human leukemia cells: evidence for drug-resistant form of topoisomerase II. *J Biol Chem* **264**: 16411–16420

 *© 2000 Cancer Research Campaign*
